# Machine Learning-Assisted Secure Random Communication System

**DOI:** 10.3390/e27080815

**Published:** 2025-07-29

**Authors:** Areeb Ahmed, Zoran Bosnić

**Affiliations:** University of Ljubljana, Faculty of Computer and Information Science, Večna pot 113, 1000 Ljubljana, Slovenia; zoran.bosnic@fri.uni-lj.si

**Keywords:** machine learning, α-stable distributions, decision tree, covert communication, random communication system

## Abstract

Machine learning techniques have revolutionized physical layer security (PLS) and provided opportunities for optimizing the performance and security of modern communication systems. In this study, we propose the first machine learning-assisted random communication system (ML-RCS). It comprises a pretrained decision tree (DT)-based receiver that extracts binary information from the transmitted random noise carrier signals. The ML-RCS employs skewed alpha-stable (α-stable) noise as a random carrier to encode the incoming binary bits securely. The DT model is pretrained on an extensively developed dataset encompassing all the selected parameter combinations to generate and detect the α-stable noise signals. The legitimate receiver leverages the pretrained DT and a predetermined key, specifically the pulse length of a single binary information bit, to securely decode the hidden binary bits. The performance evaluations included the single-bit transmission, confusion matrices, and a bit error rate (BER) analysis via Monte Carlo simulations. The fact that the BER reached 10^−3^ confirms the ability of the proposed system to establish successful secure communication between a transmitter and legitimate receiver. Additionally, the ML-RCS provides an increased data rate compared to previous random communication systems. From the perspective of security, the confusion matrices and computed false negative rate of 50.2% demonstrate the failure of an eavesdropper to decode the binary bits without access to the predetermined key and the private dataset. These findings highlight the potential ability of unconventional ML-RCSs to promote the development of secure next-generation communication devices with built-in PLSs.

## 1. Introduction

Machine learning (ML) has transformed all aspects of contemporary communication systems, such as signal processing, channel estimation, resource allocation, error correction, and security protocols. This has enhanced the efficiency, flexibility, and reliability of the current 5G and next-generation 6G systems [1,2]. Specifically, regarding signal detection and processing, model-driven deep learning networks have enhanced the performance of various channel environments for multiple-input multiple-output (MIMO) detection by optimizing the trainable parameter errors [3]. Similarly, within channel estimation, prediction, and compression, new ML approaches that are capable of adapting to changing channel conditions through online training and occasional retraining have been presented for multicarrier systems [4]. ML has also aided in the active resource management of the previous 5G spectrum and more advanced networks for radio access network (RAN) slicing [5]. In addition, the application of machine learning with a rateless scheme for error correction and decoding has established future trends in wireless communications [6]. Moreover,, the integration of ML with conventional protocols in order to optimize every component of communication systems has been extensively explored. Additionally, its ability to enhance security in preliminary open system interconnection (OSI) layers is also attracting attention with regard to future-generation security schemes.

In the above context of OSI layers, the physical layer security (PLS) is considered the front wall of any communication system. This enhances confidentiality without relying solely on cryptographic methods. It prevents unauthorized access to the first layer by leveraging the unique characteristics of the communication channels [7]. Boosting the PLS of all communication systems is the most critical requirement of the current global digital era. Recent technological advancements, new challenges, innovative solutions, and the prospects for PLS techniques with regard to the present 5G systems and upcoming 6G networks have gained considerable attention [8,9]. The security of 5G access networks has evolved over the decades, and researchers are now dedicated to the development of physical-level security architectures from scratch. This includes the use of various techniques, such as millimeter-wave communication, PLS coding, nonorthogonal multiple access, massive MIMO, and other advanced and conventional methods. It is also necessary to meet the needs of the main 6G use cases, including autonomous cyber–physical systems, machine communications, and ultralow-latency communications [10,11]. PLS has been identified as a potential solution to the underlying problems associated with traditional encryption methods, but it is considered to have several limitations [12]. As a result, its usage has diversified into other areas, such as military communications, vehicular networks, the internet of things (IoT), industrial control systems, and satellite networks [13,14,15,16,17]. These developments call for the creation of new PLS mechanisms and the assessment of previous mechanisms from different angles.

This paper contributes to the existing literature in the following ways:

- Incorporation of ML into RCSs: This paper contributes to PLS by proposing the first possible configuration of a machine learning-assisted random communication system (ML-RCS).
- Enhanced data rate and security: Our proposed user and device authentication model shows increased performance and security between a transmitter and legitimate receiver. It strengthens the PLS by covertly conveying the binary information by utilizing noise as the carrier signals. Moreover, the incorporation of an ML algorithm provides increased data rates in comparison to the previously proposed models.
- Baseline model and benchmark for comparison: The paper can be considered as presenting a baseline model for encrypting and decrypting α-stable noise signals by utilizing ML algorithms. The proposed methodology for presenting simulation results can be used as a benchmark to compare any ML-RCS proposed in the future.

The remainder of this paper is organized as follows. The theoretical background, practical utility of α-stable noise, and associated research are presented in the first part of Section 3. This is followed by a general description of decision trees and their ability to address noise signals in the second part. The design of the proposed system and some of the primary results are presented in Section 4 and Section 5. In Section 6, we review the necessary performance criteria used to evaluate RCSs. This is followed by the complete performance and analysis of the proposed ML-RCS through Monte Carlo simulations. Finally, we provide concluding remarks and directions for future research in Section 7.

## 2. Machine Learning for Physical Layer Security

ML has recently evolved into a potential candidate for fortifying PLS and achieving low-latency communication through an open radio access network architecture. It offers adaptive techniques that are based on a data-driven approach to resisting incoming threats and dynamically adjusting the defenses of 5G and 6G systems [18]. The contributions of machine learning to strengthening PLS can be categorized into three domains:

1. User and device authentication via ML.
2. Intrusion detection systems via ML.
3. Confidentiality using ML.

1. In the case of authentication, ML techniques, such as decision trees (DTs), support vector machines (SVMs), and k-nearest neighbors (KNNs), exploit physical channel features, such as channel state information and time of arrival, to achieve reliable results for MIMO systems and mobile scenarios [19]. Channel analysis [20] and signal features [21] can also help to determine the class in order to identify or detect potential attacks. This shows the versatility of ML for elevating PLS.

2. For intrusion detection, ML classifiers, such as SVMs, KNNs, DTs, and other boosting algorithms, have already been employed to perform attack identification in wireless systems, automation systems, and IoT networks [22,23,24]. Advanced techniques, including autoencoders [25] and graph neural networks [26], have also utilized temporal and multivariate features for intrusion detection. Moreover, versatile approaches have been adopted in order to identify unknown attacks on communication systems; these include simultaneous classification in address jamming [27] and incorporating perimeter intrusion detection with the generation of a curated dataset [28].

3. Specifically, to ensure confidentiality between a transmitter and legitimate receiver, the channel state information (CSI) has been exploited as a secure key in various studies. For example, eavesdropping attempts have been rebuffed by the use of secure beamforming vectors adapted to the CSI, in such a way that the original data can be recovered only by legitimate users [29]. Moreover, machine learning-based transmitter antenna selection with partial CSI (the CSI of an eavesdropper is unknown) and full CSI (the CSI of an eavesdropper is known) has been explored by deploying an SVM and naive Bayes to maximize channel secrecy [30]. Overall, ML has contributed significantly to the enhancement of PLS in all the categories discussed above.

Specifically, DT algorithms play a key role in the PLS enhancement of communication systems. They provide a versatile method for intrusion detection, channel authentication, and confidentiality. As ML algorithms, they have been used extensively in the development of underlying models that focus on data security and privacy [31]. Owing to the low computational complexity of DTs, it is widely recognized that they ensure secure communication across diverse fields, including biomedicine, mobile healthcare, power systems, automated systems, cloud computing, wireless sensor networks, and cybersecurity [32,33,34,35,36,37,38]. Less complex DTs are now being specifically designed in order to optimize the privacy and security of communication systems [39,40]. Therefore, compared with traditional static approaches, they are now considered more effective for addressing the unique challenges of PLS in communication systems. However, well-known ML algorithms, such as neural networks, naive Bayes, logistic regressions, random forests, support vector machines, decision trees, and k-nearest neighbors, have significantly improved accuracy, recall, and precision across different training datasets. However, the performance of ML algorithms incorporated within conventional communication systems begins to diminish after a certain security benchmark is achieved [41]. This stresses the vulnerabilities of ML approaches when they are incorporated into conventional communication systems. Moreover, it is necessary to explore the possibility of incorporating ML into unconventional communication systems to enhance the PLS.

Among the various unconventional communication mechanisms, the utilization of noise as a random carrier can be integrated into ML algorithms. Specifically, if secure communication must be established in the additive white Gaussian noise (AWGN) channel, alpha-stable (α-stable) noise can provide coverage by exploiting its non-Gaussian distribution characteristics. In general, it has been used to model channels that exhibit heavy tails, skewed data, and responses that are vulnerable to unpredictable fluctuations. However, its ability to covertly convey binary information to legitimate receivers has also been exploited. The key advantage of utilizing α-stable noise as a carrier is twofold: first, it safeguards encrypted binary information, and second, it masks the presence of communication [42]. Random communication systems (RCSs) have initiated the utilization of symmetric α-stable (SαS) and skewed α-stable (SkαS) noise as random carrier signals to establish covert communication between a transmitter and legitimate receiver [43,44]. Both schemes utilize static estimation methods, such as the sinc estimator and logarithmic estimator, to decrypt the transmitted random carrier signals at the legitimate receiver by utilizing a predetermined pulse length. The most refined design was later proposed on the basis of the maximum extreme value method-based estimator [45]. This provided an optimized version of an RCS with respect to the bit error rate. This was followed by the development of the first synchronization method for RCSs, paving the way for their practical implementation in real-world applications, such as military communications, vehicular networks, the internet of things, industrial control systems, and satellite networks [46]. Studies have also been conducted to expand the symbol capacity by introducing multiple-level M-ary modulation schemes [47]. This resulted in an increase in the data rate achievable by RCSs. The PLS achievable by RCSs is theoretically guaranteed due to the nonexistence of higher-order statistical properties in α-stable noise. This results in an inherent resistance to eavesdropping as a random carrier. Therefore, the initially proposed architectures focused on improving the bit error rate (BER). Nevertheless, several RCS architectures concatenated with other entities, such as inverse systems and signal multiplexers, were later proposed [48,49]. The primary objective of these studies was to extend the parameters or private keys from a single parameter, that is, the pulse length, to multiple parameters. This was because these parameters or private keys govern the coverage of RCSs. The approach of deploying various static estimators along with the integration of several entities in an RCS has improved the performance of the BER and security [50]. On the other hand, the practicality of RCSs for the internet of things has also been tested, which has strengthened its potential for real world applications [51]. However, there is still a significant gap between conventional communication systems and α-stable noise-based unconventional RCSs in terms of the data rate scale. ML algorithms could play a key role in reducing this gap. This could further prove their applicability for strengthening the PLS of future 6G nano-communication systems if they can compete with conventional communication in terms of the data rate [52].

Considering these factors, we propose a novel machine learning-assisted secure random communication system (ML-RCS). This is the first attempt to deploy any ML algorithm to encrypt and decrypt the α-stable noise at a transmitter and legitimate receiver, respectively. The proposed method belongs to the category of ML-based user and device authentications. The proposed ML-RCS establishes covert communication in the AWGN channel and strengthens the PLS of the system. The transmitter ‘T’ generates SkαS noise as a random carrier on the basis of the incoming binary bits. The inherent symmetry of the skewness parameter of the SkαS noise is exploited to transmit binary bits ‘0’ and ‘1’. The legitimate receiver ‘LR’ uses the least complex ML algorithm, that is, a DT, which is pretrained on a developed α-stable noise signal model (α-SNSM). The α-SNSM comprises all the combinations of parameters required to generate the SkαS noise as a random carrier. Moreover, we include a unique private key, that is, the pulse length, in the α-SNSM while training the DT-based receiver, and we assume that it is known only to the T and LR. Only the LR can decrypt the transmitted SkαS noise and retrieve the binary information via the pretrained DT and a unique private key. However, no eavesdropper can decrypt the transmitted SkαS noise and retrieve the binary information without knowledge of the pretrained DT and the unique private key. Integrating ML into an RCS in this unique way produces an extremely efficient BER performance and enhanced covertness in comparison with previous RCSs, which were based on static estimators. The proposed ML-RCS can retrieve a unit binary bit from transmitted random carrier signals by utilizing an extremely small number of SkαS noise samples. Hence, it provides a significantly increased data rate in comparison with previously proposed RCSs.

The reason for choosing DTs is their probable suitability as receivers operating with alpha-stable noise since they have a non-parametric nature and provide robustness against outliers. Opposite to the prominent ML algorithms (KNNs, SVMs, and neural networks), DTs do not rely on distance metrics or statistical assumptions due to their absence under heavy-tailed alpha-stable noise. They have been shown to operate with nonlinear decision boundaries in a good manner. Most importantly, they require minimum computational complexity for real-time applications, making them a feasible choice for signal classification in non-Gaussian noise conditions. Therefore, we expect DTs to be a more practical choice with which to establish a baseline ML-RCS model which can be used as a benchmark in the future. Their use makes the ML-RCS a potential candidate for providing security for future communication systems in which security through unconventional mechanisms is explicitly needed.

## 3. Allied Concepts

In this study, we investigate the integration of an ML algorithm with an RCS; it is therefore necessary to delve into the principles of the methods used. The proposed ML-RCS begins by harnessing the antipodal characteristics of the SkαS noise carrier signals for transmitting binary bits. Therefore, in this section, we first discuss the general structure of the α-stable noise and its special cases. We train the data retrieved via the LR using the DT. To better understand the training and decision-making phases of DTs, we present them in detail and explain the variants of DTs utilized. We then explore the DT decisions obtained when dealing with the α-stable noise signals.

### 3.1. Alpha Stable (α-Stable) Distribution

α-stable noise, a well-known random noise model, has been generally identified as a severe channel impairment in communication systems because of its unique properties. In contrast to AWGN, the heavy-tailed distribution of α-stable noise can cause significant disruption to communication channels. α-stable noise simulates impulsive channel conditions. It is highly relevant in contexts such as vehicular networks, space communications, wireless systems, and IoT scenarios, which are susceptible to excessive interference [53,54,55].

1. The α-stable distribution includes several subcategories of α-stable noise: symmetric α-stable (SαS) and skewed α-stable (SkαS). The overall statistical parameters of the distribution are denoted by $S_{\alpha }$ (*β*, γ, *μ*), where a random variable R follows R~$S_{\alpha }$ (*β*, γ, *μ*). The parameters responsible for defining the distribution are as follows.
2. The characteristic exponent (α) controls the impulsiveness of the distribution and varies within α ∈ (0, 2].
3. The skewness (β) governs the asymmetry of the distribution, with *β* = −1 creating a leftward skew and *β* = 1 creating a rightward skew, falling within β∈ [−1, 1].
4. The scale parameter (γ) is the dispersion or scaling of the distribution within γ ∈ (0, ∞).
5. The location parameter (μ) shifts the distribution along the horizontal axis with μ ∈ (−∞,∞).

The basic structure of the α-stable noise R~$S_{\alpha }$ (*β*, γ, *μ*) is dependent on its characteristic function derived in [56]:


(1) $$\phi \left(\theta \right)=\left\{\begin{array}{c} exp\left\{j\mu \theta -\gamma^{\alpha \;}{\left|\theta \right|}^{\alpha }\left(1-j\beta sign\left(\theta \right)tan\left(\frac{\alpha \pi }{2}\right)\right)\right\};\;if\;\alpha \neq 1\\ exp\left\{j\mu \theta -\gamma \;\left|\theta \right|\left(1+j\beta \frac{2}{\pi }sign\left(\theta \right)ln\left(\frac{\alpha \pi }{2}\right)\right)\right\};\;if\;\alpha =1 \end{array}.\right\}$$

**Figure 1:**
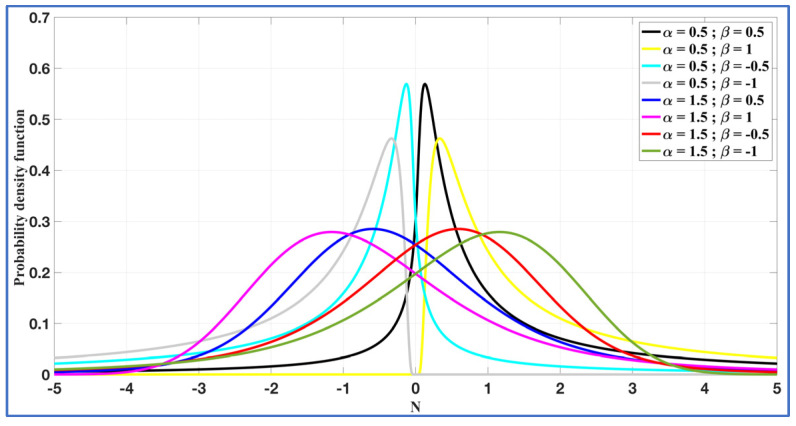
PDF of α-stable noise for parameters α and β, where γ = 1.0 and µ = 0.

In this study, α-stable noise was produced via the approach described in [57]. A range of probability density functions (PDFs) for R~$S_{\alpha }$ (*β*, γ, *μ*) were produced, as illustrated in Figure 1, by systematically varying the parameters α and β within their respective ranges while keeping μ=0 and γ = 1 constant. This was performed to understand the basic roles of α and β in dealing with α-stable noise.

**Figure 2:**
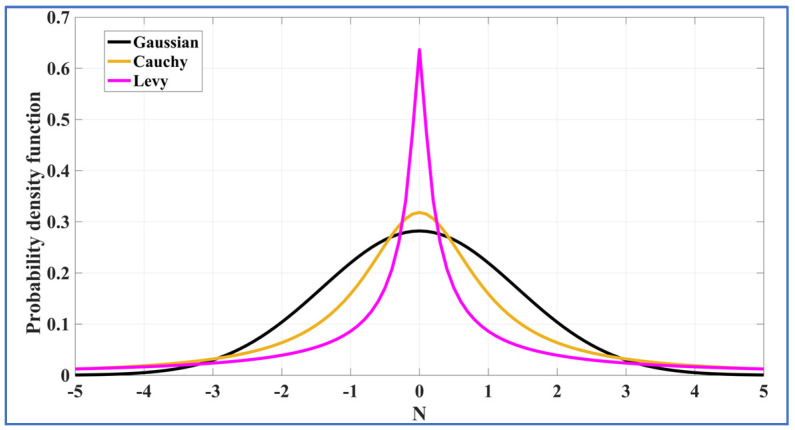
PDF of α-stable noise for various special noises.

Note: Specific cases within the α-stable family, such as R~$S_{\alpha =2}$ (*β* = 0, γ, *μ*), R~$S_{\alpha =1}$ (*β* = 0, γ, *μ*), and R~$S_{\alpha =0.5}$ (*β* = 1, γ, *μ*), represent Gaussian, Cauchy, and Levy noises, respectively, as shown in Figure 2. In contrast, SkαS noise is represented by R~$S_{\alpha }$ (+*β*/−*β*, γ, *μ*), and its characteristics are illustrated in Figure 1.

When α < 2, the corresponding distribution has a second-order moment-generating function. However, a first-order moment-generating function does not exist under such conditions. As shown in [56,57], α-stable noise accurately models heavy-tailed impulsive events that are orders of magnitude larger than the channel impairments induced by AWGN. This characteristic makes it virtually undetectable by illegitimate receivers or eavesdroppers, while also conferring natural immunity to channel distortions. Consequently, α-stable noise is an appealing candidate for physical layer covert communications.

### 3.2. Decision Trees

DTs enable one to conveniently perform simple and intelligent decision-making within complex processes by dividing them into smaller and manageable components. This enables straightforward interpretation, which represents a powerful yet easy way to analyze several variables. Several approaches are available for DT construction. However, C4.5 [58], the enhanced version of iterative dichotomizer 3 (ID3), and classification and regression trees (CARTs) [59], are considered the most reliable algorithms when decision-making on complex data is required through a single tree. If the two algorithms are compared on the basis of accuracy, the C4.5 algorithm is more suitable for the construction of DTs with datasets that possess negligible or no noise. In contrast, the performance of CART algorithms has been proven to be better for noisy datasets [60]. It has been found to be comparatively successful, particularly in the presence of random noise in a dataset. Moreover, its accuracy has been extensively tested on standardized datasets that have already added random noise as a class variable with varying noise power. It has also proven its applicability to datasets containing label noise during the training phase of supervised classification [61]. Specifically, when conventional communication systems are incorporated, a CART significantly enhances the classification accuracy compared with static methods. It has also shown robustness in detecting and estimating mixed noise signals in challenging communication environments, such as fading channels [62]. This makes the CART approach a potential candidate for the efficient estimation of SkαS noise carrier signals for RCSs. Because this is an underlying principle that enables the retrieval of binary information via the LR in the proposed ML-RCS, we proceed by reviewing the basic methodology behind it.

#### CART Classifier Algorithm

**Figure 3:**
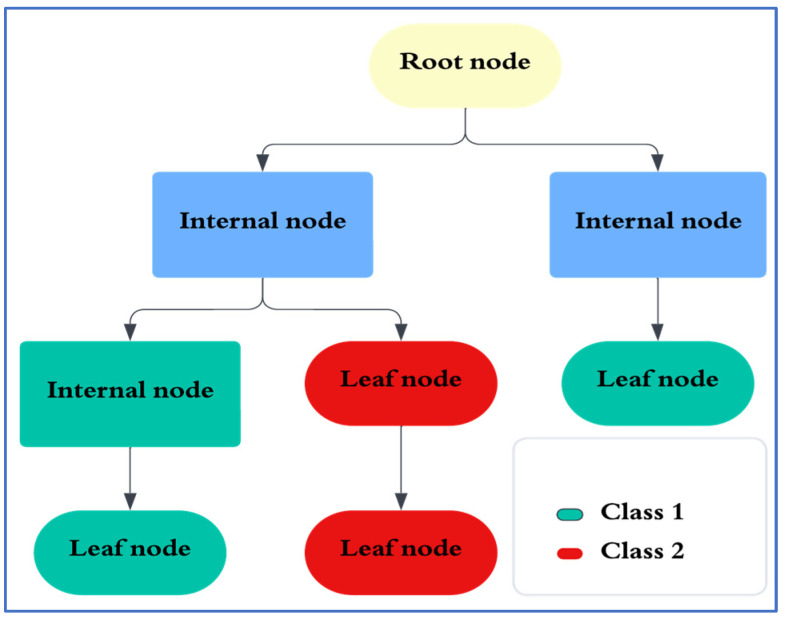
Basic structure of the decision tree.

The CART was introduced by Breiman et al. in 1984 [59]. It uses a binary splitting mechanism to construct DTs. The splits are evaluated on the basis of the Gini index (*GI*). It is used to compute the impurities of a particular node. The *GI* is the deciding factor in selecting the splitting attribute variable. For a dataset D, the *GI* is computed as follows:

(2) $$\mathrm{GI}\;\left(D\right)=1-\sum_{i=1}^{m}p_{i}^{2}$$

where the *i-th* class proportion of instances is denoted by ‘$p_{i}$’ among the total ‘*m*’. A value of ‘*GI* = 0’ reflects a perfectly pure node, whereas equal classes reflect the maximum impurity. The *GI* of the split ‘*GIS*’ is given by

(3) $$\mathrm{GIS}\;(D)=\frac{n_{1}}{n}\;\mathrm{GI}\;\left(D_{1}\right)+\frac{n_{2}}{n}\;\mathrm{GI}\;\left(D_{2}\right)$$

during the splitting of dataset D into subsets $D_{1}$ and $D_{2}$. The subsets $D_{1}$ and $D_{2}$ are of sizes ‘$n_{1}$’ and ‘$n_{2}$’, respectively. The ‘n’ represents a size of D. To classify a specific class, the CART algorithm proceeds with the split that attains the minimum *GI* for the subsequent subsets. The basic structure of the DT is shown in Figure 3.

Note: The topmost node in the tree is the root node representing the entire dataset. All the nodes are split on the basis of the attribute variable, resulting in branches and internal nodes. A node that does not split further is known as a leaf node. The leaves represent the final decisions of the tree and contain the target value as the predicted outcome, which is referred to as the ‘class’.

CART Classifier Variations: Fine tree (FT), coarse tree (CT), and medium tree (MT) are variations of DTs; these differ in their stopping criteria when training a tree by recursively splitting the data. Table 1 summarizes the three variations used in this study.

## 4. Transmission

**Figure 4:**
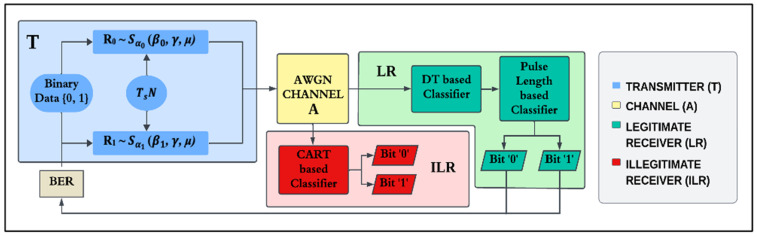
Model of the proposed ML-RCS for two scenarios: (1) transmitter and legitimate receiver in AWGN channel, and (2) transmitter and illegitimate receiver in AWGN channel.

The proposed ML-RCS model is shown in Figure 4. It consists of a transmitter ‘T’, legitimate receiver ‘LR’, and illegitimate receiver ‘ILR’. The ML-RCS operates in the AWGN channel. Unlike the ILR, the T and LR are assumed to be perfectly synchronized by the method given in [46] and have predetermined pulse lengths and parameters, which are utilized to generate the SkαS noise as random carriers. In contrast, the ILR is used to analyze the covertness of the ML-RCS from the perspective of an eavesdropper. To depict the worst-case scenario in this context, the ILR can presumably operate via the same CART-based classifier. However, it does not have knowledge of the predetermined pulse length or the parameters utilized by the T and LR.

### Transmitter ‘T’

The transmitter ‘T,’ shown in Figure 4, operates on the principle of stochastic process shift keying or noise shift keying (NSK). It transmits the incoming binary information, namely ‘0’ and ‘1’, by generating R_0_~$S_{\alpha_{0}}$ ($\beta_{0}$, γ, *μ*) and R_1_~$S_{\alpha_{1}}$ ($\beta_{1}$, γ, *μ*) noises as random carrier signals. Because $\beta_{1}=\;{-\beta }_{0}$, the symmetry behind the skewness parameters ‘$\beta_{0}$’ and ‘$\beta_{1}$’ are exploited to shift the SkαS noise distributions to either the left or right side, respectively. This is intentionally performed to generate noise samples from distributions $S_{\alpha_{0}}$ ($\beta_{0}$, γ, *μ*) and $S_{\alpha_{1}}$ ($\beta_{1}$, γ, *μ*) to represent the corresponding ‘0’ and ‘1’ values. The term $∆\beta ≜\;\beta_{1}-\;{(\beta }_{0})$ is used to monitor the difference between $\beta_{0}$ and $\beta_{1}$. The samples are generated as per the predetermined amount, which is known as the pulse length ‘$T_{s}N$’ or simply N, as $T_{s}=1$ is considered throughout this study. It is necessary to encode a unit bit as ‘1’ or ‘0’ in the following form:

(4) $$R\;=\;\{r_{1},\;r_{2},\;\ldots ,\;r_{N}\}$$

where N is the total generated noise sample of length $T_{s}$, which corresponds to a single binary bit. The binary information ‘0’ and ‘1’, hidden in random carriers R_0_~$S_{\alpha_{0}}$ ($\beta_{0}$, γ, *μ*) and R_1_~$S_{\alpha_{1}}$ ($\beta_{1}$, γ, *μ*), respectively, cannot be retrieved without an exact knowledge of $T_{s}N$ [46,50]. This method is known as skewed α-stable noise shift keying (SkαSNSK). The complexity of R_0_~$S_{\alpha_{0}}$ ($\beta_{0}$, γ, *μ*) and R_1_~$S_{\alpha_{1}}$ ($\beta_{1}$, γ, *μ*) can be further increased by selecting different characteristic exponents $\alpha_{0}$ and $\alpha_{1}$. However, they are kept the same, as $\alpha_{0\;}=\alpha_{1}=\;\alpha$, throughout the study to analyze the effect of only hiding the binary information on the skewness parameters. Similarly, the scale and location parameters are predetermined between the T and LR, as γ=1 and μ=0, respectively. Consequently, the factors responsible for generating unique random carriers in different settings are α, ∆β, and N.

Any specific values of α, ∆β, and N, within their defined ranges, must be selected in advance by the T and LR to securely transmit the hidden binary information via SkαSNSK. These values act as secure keys in the data retrieval process via the LR. In this study, they are set to the values listed in Table 2.

The values of α and ∆β, as listed in Table 2, are typically used while experimenting with α-stable noise signals. However, the values of N were derived after rigorous experimentation with the values used in [43,44,45,46,47,48,49,50]. During the experiments, we examined various combinations of the above parameter values to analyze the effects on the performance of ML-RCSs for the T and LR; we also aimed to determine a trend in the performance while exhaustively searching the parameters responsible for SkαSNSK.

## 5. Reception

The proposed ML-RCS receiver works in the AWGN channel, and the transmitted SkαSNSK noise sequences R_0_~$S_{\alpha }$ ($\beta_{0}$, γ, *μ*) and R_1_~$S_{\alpha }$ ($\beta_{1}$, γ, *μ*), that is, **R** or the output of the T, travel through it. Therefore, the functionality of AWGN is first explained before proceeding to the LR.

### 5.1. AWGN Channel

The AWGN channel **A**, as shown in Figure 4, distorts the transmitted noise sequences via the distribution given below

(5) $$A\;\sim \;S_{\alpha_{A=2}}(\beta_{A}=0,\;\gamma_{A},\;\mu_{A}\;=\;0)$$

The impairments introduced by **A** are similar to the random characteristics inherently built into **R**. Hence, the conventional criterion ‘signal-to-noise ratio’ is not applicable for measuring the performance of unconventional RCSs. Therefore, the effects of channel impairments on **R** are usually analyzed via the criteria of the dispersion ratio, scale ratio, or mixed signal-to-noise ratio (MSNR). This was introduced in [63] as follows:

(6) $${\mathrm{MSNR}}_{\mathrm{dB}}\;=10\log\frac{\gamma }{\gamma_{A}}$$

Using (6), the ML-RCS can be analyzed under various channel settings by changing $\gamma_{A}$ to imitate the noise variants encountered by **R** while propagating through the AWGN channel. The ranges of $\gamma_{A}$ and the corresponding MSNRs utilized in this study are summarized in Table 3.

### 5.2. Legitimate Receiver ‘LR’

The legitimate receiver LR receives noise sequence **Y** as

**Y** = **R** + **A** (7)

which is accessible to both the LR and the ILR. As defined in Figure 4, the LR first performs a cart-based classification, followed by a pulse length-based classification, to retrieve a unit binary bit that is hidden in **Y**. The received noise sequence **Y** carrying the unit binary bit is defined as

(8) $$Y=\{y_{1},\;y_{2},\;\ldots ,\;y_{N}\}$$

#### 5.2.1. DT-Based Classifier

A cart-based classification is performed by utilizing a pretrained DT that has been trained on an α-stable noise signal dataset (α-SNSD) solely known to the T and LR. The α-SNSD used for training the DT consists of a random carrier signal and channel settings. The target variable to be predicted is the skewness parameter of the α-stable noise carrier. The utilized α-SNSD is expressed as

(9) $$D=\{(\alpha ,\;\beta ,\;N,\;{\mathrm{MSNR}}_{\mathrm{dB}},\;y)\}$$

where α ∈ {0.6, 0.8, 1.1}, β ∈ {−1, −0.9, −0.8, 0.8, 0.9, 1}, and N ∈ {15, 25, 35}, as defined in Table 2. The MSNR_dB_ ∈ [−10, 5] is defined in Table 3. The ‘*y*’ is the α-stable noise sample generated via the specified combination of α, β, N, and MSNR_dB_. Each unique combination in (9) produces a different ‘*y*’ each time, so 100 unique ‘*y*’ values are taken to train the DT for a single combination. The DT-based classifier is trained on an α-SNSD to predict the target parameter $\hat{\beta }$. The classifier learns to predict the output as follows:

(10) $${\hat{\beta }}_{t}=\;f(\alpha ,\;N,\;{\mathrm{MSNR}}_{\mathrm{dB}},\;y_{t})$$

#### 5.2.2. Pulse Length-Based Classifier

For legitimate communication between the T and LR, the DT is trained on an α-SNSD, which consists of three different values of N. Therefore, any one value of N can be prechosen while carrying out secure communication. Specifically, from the perspective of the LR, the predicted parameter values {${\hat{\beta }}_{\;1},\;{\hat{\beta }}_{2},\;\ldots ,\;{\hat{\beta }}_{N}$} carrying a single binary bit are then provided to the pulse length-based classifier. It classifies the incoming {${\hat{\beta }}_{\;1},\;{\hat{\beta }}_{2},\;\ldots ,\;{\hat{\beta }}_{N}$} carrying a unit bit to a single classified beta ‘$\beta_{C}$’ using the following relation:

(11) $$\beta_{C}=\;\left\{\begin{array}{c} \beta_{0}\;;if\;\sum_{t=1}^{N}{\hat{\beta }}_{t}<0\\ \beta_{1};if\;\sum_{t=1}^{N}{\hat{\beta }}_{t}\geq 0 \end{array}\right\}$$

where $\beta_{0}$ represents the retrieval of the bit as ‘0’ and where $\beta_{1}$ represents the retrieval of the bit as ‘1’. The procedure is repeated to retrieve all subsequent binary bits.

### 5.3. Demo Transmission

**Figure 5:**
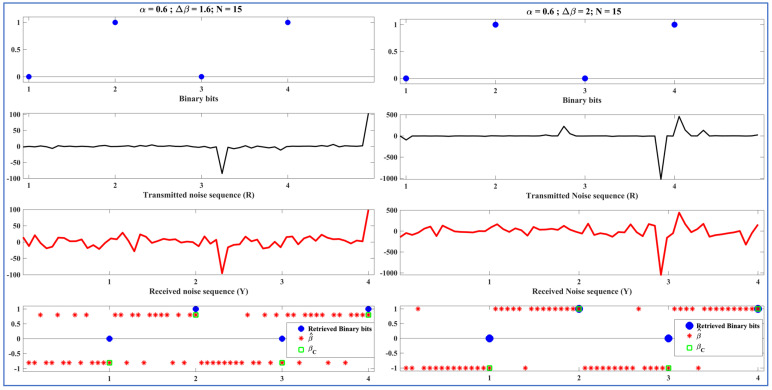
Left diagram: Binary bits and transmitted noise sequence (R) at T (top two), received noise sequence (Y), classified beta $\beta_{C},$ and corresponding retrieved binary bits at LR (bottom two); α = 0.6, ∆β = 1.6, N = 15. Right diagram: Binary bits and transmitted noise sequence (R) at T (top two), received noise sequence (Y), classified beta $\beta_{C}$, and corresponding retrieved binary bits at LR (bottom two); α = 0.6, ∆β = 2, N = 15.

**Figure 6:**
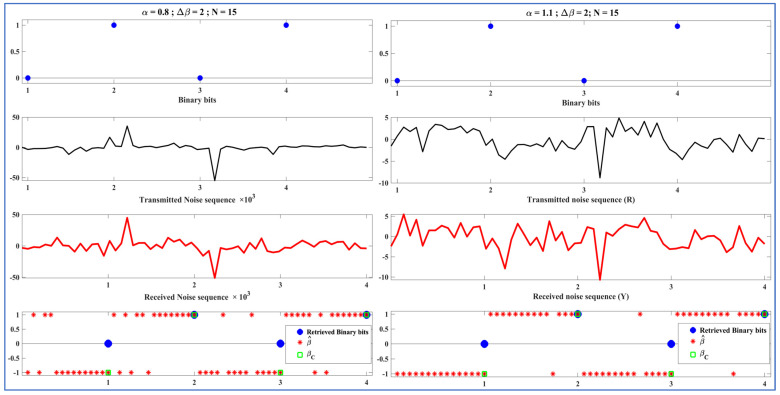
Left diagram: Binary bits and transmitted noise sequence (R) at T (top two), received noise sequence (Y), classified beta $\beta_{C}$, and corresponding retrieved binary bits at LR (bottom two); α = 0.8, ∆β = 2, N = 15. Right diagram: Binary bits and transmitted noise sequence (R) at T (top two), received noise sequence (Y), classified beta $\beta_{C}$, and corresponding retrieved binary bits at LR (bottom two); α = 1.1, ∆β = 2, N = 15.

**Figure 7:**
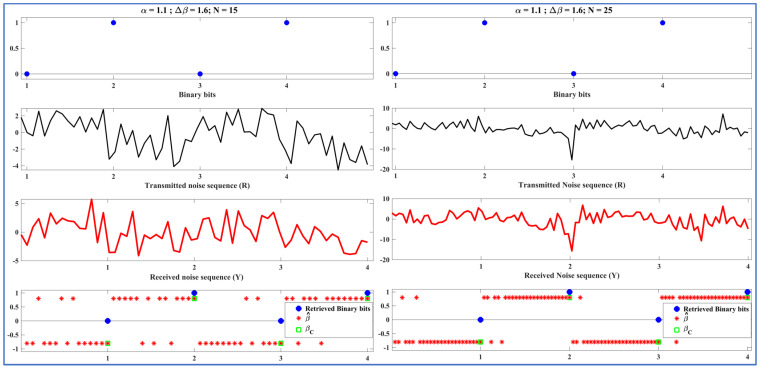
Left diagram: Binary bits and transmitted noise sequence (R) at T (top two), received noise sequence (Y), classified beta $\beta_{C}$, and corresponding retrieved binary bits at LR (bottom two); α = 1.1, ∆β = 1.6, N = 15. Right diagram: Binary bits and transmitted noise sequence (R) at T (top two), received noise sequence (Y), classified beta $\beta_{C}$, and corresponding retrieved binary bits at LR (bottom two); α = 1.1, ∆β = 1.6, N = 25.

Before analyzing the performance of the ML-RCS on a large number of binary bits, we tested it on fewer bits to better represent and understand the signals processed by the T and LR. The demo transmissions in this section demonstrate the proof-of-concept. In Figure 5, Figure 6 and Figure 7, the communication consists of incoming binary bits and the corresponding noise sequence ‘R’ transmitted from the perspective of the T. This sequence is further followed by the received noise sequence ‘Y,’ which is predicted by $\hat{\beta }$ and computed as $\beta_{C}$ to retrieve binary bits from the perspective of the LR. We present the initial test of the binary stream of bits ‘0,1,0,1’ in Figure 5, Figure 6 and Figure 7. In this section, we vary one of the SkαSNSK factors responsible for the SkαSNSK (α, ∆β, and N) simultaneously. The other two parameters are fixed. We conducted this study to examine the trends for all possible combinations.

Figure 5, Figure 6 and Figure 7 show that the retrieval process at the T and LR improved with increasing the SkαSNSK responsible factors (α, ∆β, and N). Specifically, the predicted parameter values {${\hat{\beta }}_{\;1},\;{\hat{\beta }}_{2},\;\ldots ,\;{\hat{\beta }}_{N}$} that carried a single binary bit had fewer errors when greater values of α, ∆β, and N were utilized by the T and LR.

Figure 5 shows the transmitted noise sequence obtained by varying ∆β while keeping the other two parameters fixed. Figure 5 (left diagram) represents the transmission with ∆β = 1.6, where the T and LR utilize $S_{\alpha =0.6}$ ($\beta_{=0.8}$, γ=1, μ = 0) for binary ‘1’ and $S_{\alpha =0.6}$ ($\beta_{=-0.8}$, γ=1, μ = 0) for binary ‘0’. However, in Figure 5 (right diagram), with ∆β = 2, we use the random carriers $S_{\alpha =0.6}$ ($\beta_{=1}$, γ=1, μ = 0) and $S_{\alpha =0.6}$ ($\beta_{=-1}$, γ=1, μ = 0) for binaries ‘1’ and ‘0,’, respectively. Figure 5 and Figure 6 indicate that increasing ∆β from 1.6 to 2 results in fewer errors for the predicted $\hat{\beta }$. This indicates a gradual increase in the BER performance of the ML-RCS with an increased ∆β.

Similarly, we vary α in Figure 6 while keeping ∆β and N fixed to observe the trend. Specifically, in Figure 6 (left diagram), the T and LR utilize the following parameters: $S_{\alpha =0.8}$ ($\beta_{=1}$, γ=1, μ = 0) for binary ‘1’ and $S_{\alpha =0.8}$ ($\beta_{=-1}$, γ=1, μ = 0) for binary ‘0’. However, in Figure 6 (right diagram), we use $S_{\alpha =1.1}$ ($\beta_{=1}$, γ=1, μ = 0) and $S_{\alpha =1.1}$ ($\beta_{=-1}$, γ=1, μ = 0) for binaries ‘1’ and ‘0’, respectively. As explained previously, in Figure 6, increasing α from 0.8 to 1 has the same effect on the performance. The overall BER performance was expected to complement the results obtained in this study.

Finally, in Figure 7, we vary N while α and ∆β remain constant to analyze the potential difference in the retrieval process at the T and LR. Figure 7 (left diagram) shows the random carriers $S_{\alpha =1.1}$ ($\beta_{=0.8}$, γ=1, μ = 0) for binary ‘1’ and $S_{\alpha =1.1}$ ($\beta_{=-0.8}$, γ=1, μ = 0) for binary ‘0’, with N = 15. However, we selected N = 25, as shown in Figure 7 (right diagram). In conclusion, increasing N yields a better prediction for $\hat{\beta }$. A better prediction helps achieve better BER rates, which is also confirmed in the next section of our paper.

The successful demo transmission reflects the ability of ML-RCSs to securely convey binary information. Moreover, the trend observed in Figure 5, Figure 6 and Figure 7 was also observed for many bits in the proposed ML-RCS. The next section focuses on this issue.

## 6. Results and Discussion

Following the results and trends observed in the effective demo transmissions, in this section, we analyze the proposed ML-RCS for a larger number of bits. The generalized method of checking 1000 binary bits for errors [43,44,45,46,47,48,49,50,51,52] was adopted to compute the confusion metrics and BER performance.

First, the proposed ML-RCS was analyzed from the perspective of the T and LR, termed ‘intended communication’. This indicates that the T and LR are synchronized and in harmony with the user and device authentication. This is followed by the perspective of the T and ILR, which is referred to as ‘unintended communication’. This indicates that the T and LR are synchronized but not in harmony with the user and device authentication.

### 6.1. Intended Communication

Because the proposed ML-RCS is the first approach to incorporate ML into RCSs, we tested it using methods commonly employed to perform individual analyses in both fields. First, we analyzed the ML-RCS via confusion matrices for clarity with respect to ML. After showing successful results with the confusion matrices, we conducted Monte Carlo simulations that targeted an ideal BER of 10^−3^ against the lowest value of the MSNR to reflect its effectiveness with respect to RCSs.

#### 6.1.1. Confusion Matrices

**Figure 8:**
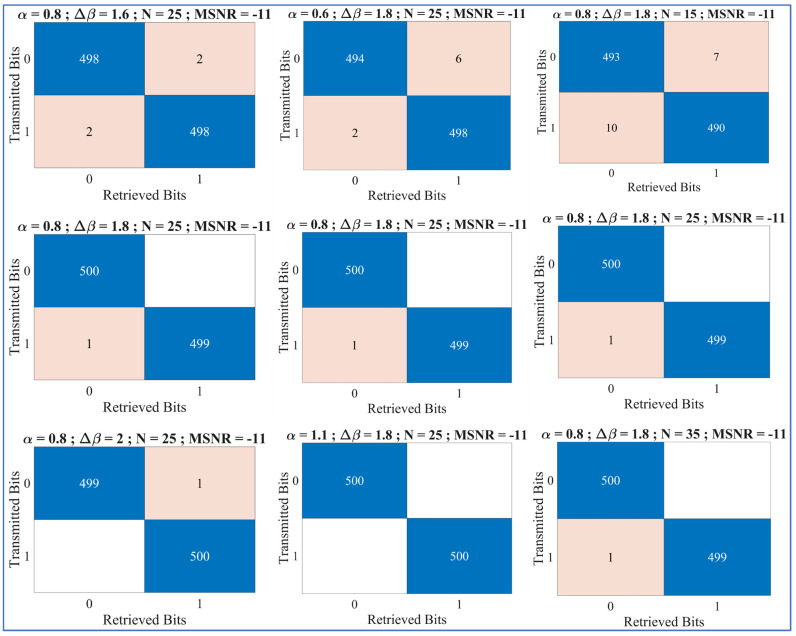
Left column: Confusion matrices when ∆β = 1.6, 1.8, and 2. This figure shows the reduction in the error during the retrieval when ∆β is increased. Middle column: Confusion matrices when α  = 0.6, 0.8, and 1.1. This figure shows the reduction in error during the retrieval when α is increased. Right column: Confusion matrices when N = 15, 25, and 35. This figure shows the reduction in the error during the retrieval when N is increased.

We compute the confusion matrices for the ‘transmitted bits’ against the ‘retrieved bits’ in Figure 8. The established trend of exhaustively varying one of the SkαSNSK responsible factors (α, ∆β, and N) and keeping the others fixed was also adopted while analyzing the ML-RCS with confusion metrics. The MSNR was constant throughout the confusion matrix.

Firstly, as shown in Figure 8 (left column), ∆β varies from 1.6 to 2. Similar to the demo transmission, the expected increase in performance is also observed in the confusion matrix as ∆β increases, as shown in Figure 8 (left column). Second, as shown in Figure 8 (middle column), α is changed from 0.6 to 1.1 while keeping ∆β and N fixed. Given the results of the demo transmission, we observed a similar increase in the performance of the confusion matrices for α. Finally, similar effects were also observed when varying N while keeping α and ∆β constant; this is also shown in Figure 8 (right column). The performance appeared to increase with every increment in N from 15 to 35.

Compared with the successful results obtained during the demo transmissions for a few transmitted bits, the previous results show that the ML-RCS achieves a favorable performance for many bits. The confusion matrices derived for all the SkαSNSK responsible factors (α, ∆β, and N) also reflect similar trends in the performance of the ML-RCS with increasing values of α, ∆β, and N, resulting in fewer errors between the transmitted and retrieved bits.

#### 6.1.2. Bit Error Rate Analysis

**Figure 9:**
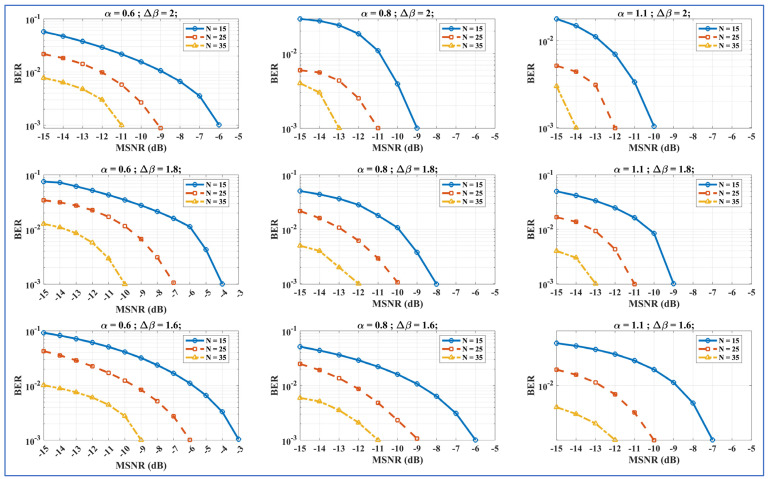
BER vs. MSNR_dB_ performance of ML-RCS; total transmitted bits = 1000. The figures show that the ML-RCS performance increases with an increment in the SkαSNSK responsible factors (α, ∆β, and N).

We checked the trends that developed for the SkαSNSK responsible factors (α, ∆β, and N) by exhaustively examining the performance of the ML-RCS with respect to the criteria pertinent to RCSs. We generated BER vs. MSNR_dB_ graphs by utilizing the same ranges of α, ∆β, and N as those used for generating the confusion matrices. In Figure 9, we present a systematic BER analysis of the ML-RCS with respect to RCSs. The simulations comprised 10,000 iterations for a single BER data point at a given MSNR_dB_. Stabilization was achieved at a tolerance of ±0.01 for 8000 iterations to verify convergence.

In Figure 9, the effects of increasing α move horizontally for a given row, whereas the effects of increasing ∆β move vertically for a given column. Moreover, every individual BER vs. MSNR_dB_ graph shows the effects of increasing N for fixed values of α, and the lowest value of the MSNR is considered an ideal performance. The figures reveal that, similar to the results in the previous sections, the performance of the ML-RCS increases as α increases for a fixed ∆β and N in any given row. Moreover, increasing ∆β, for a fixed α and N, also has an incremental effect on the BER performance. For any individual BER vs. MSNR_dB_ graph, the performance increases as N increases. The ideal BER performance is achieved at MSNR_dB_ = −14 for α = 1.1, ∆β = 2, and N = 35, which is a combination of the maximum values utilized for these parameters. This further confirms the trends found for α, ∆β, and N while exhaustively examining the performance of the ML-RCS, as observed in the previous section. Given the BER vs. MSNR_dB_ results for the previously proposed RCSs [43,44,45,46,47,48,49,50,51,52], the ideal performance of the ML-RCS is comparatively achieved against a very low value of the MSNR_dB_. This further shows that the incorporation of pretrained DTs and an α-SNSD not only inherits the building security of an SkαSNSK-based RCS, but also outperforms the previously proposed models in terms of its BER performance.

**Figure 10:**
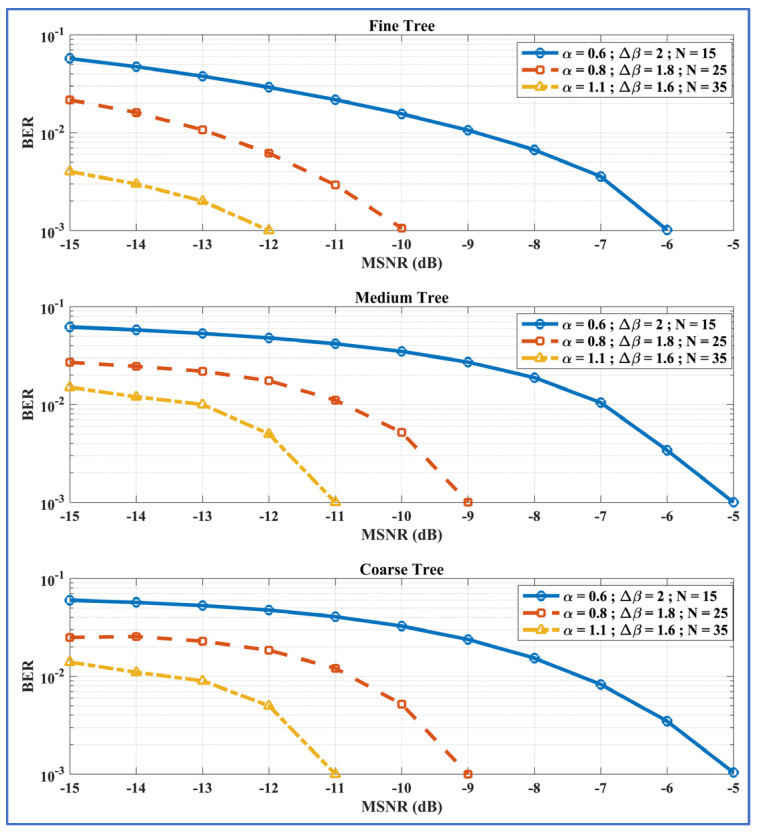
BER vs. MSNR_dB_ performances for the FT, MT, and CT configurations of the ML-RCS; total transmitted bits = 1000. The figure shows that the FT is the best configuration for the cart classifier while retrieving binary information from the SkαSNSK.

In this study, all the results for the ML-RCS were generated via an FT-based pretrained DT. However, we present a comparative BER vs. MSNR_dB_ analysis with CT-based pretrained DTs and MT-based pretrained DTs in Figure 10. We present this to justify the selection of the FT-based pretrained DT for the ML-RCS against other potential configurations. For any common combination of α, ∆β, and N, the ML-RCS achieved an ideal BER of 10^−3^, which was achieved against the lowest value of the MSNR, while utilizing the FT configuration compared with its counterparts. Therefore, the FT-based pretrained DT was chosen to record all the results in the previous sections.

### 6.2. Unintended Communication

We also analyzed the ML-RCS from the perspective of an ILR to determine its strength against potential eavesdropping. In this section, we model the ILR as a highly capable and sophisticated eavesdropper by depicting the worst-case scenario for the T and LR. As shown in Figure 4, we assume that the ILR utilizes the same DT-based classifier. However, the ILR does not have access to the α-SNSD to produce a pretrained DT similar to that developed by the T and LR for the intended communication. Therefore, the parameter settings of the SkαSNSK responsible factors (α, ∆β, and N) for the ILR are not the same as those in Table 2.

#### Confusion Matrices

The parameter settings for the ILR are given below in Table 4.

**Figure 11:**
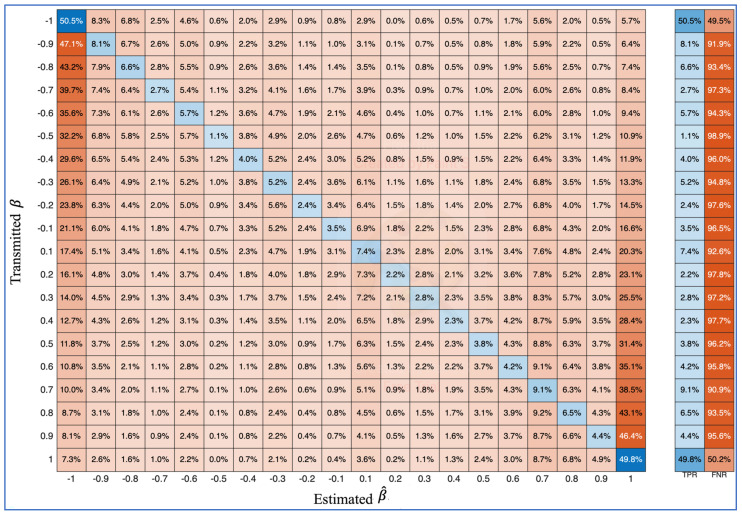
Confusion matrix for the ILR; α = 1.1, N = 26, and MSNR_db_ = −11. This figure shows the inability of the ILR to retrieve binary information.

It is important to note in Table 2 that the ILR must possess the actual pulse length (N) utilized by the T and LR to successfully retrieve the binary information hidden in the transmitted SkαSNSK signals. This is practically impossible, with a probability of $\frac{1}{\infty }$. However, to analyze the ML-RCS under the worst-case scenario in this study, the ILR is assumed to use a pulse length of ‘N + 1’, which is identical to the actual pulse length N. With the above assumption, a confusion matrix for the ILR is generated, as shown in Figure 11. Therefore, the ILR cannot correctly predict $\hat{\beta }$ even in the most favorable scenario for unintended communication. Even when ∆β = 2, the true positive rate (TPR) for $\hat{\beta }$ does not exceed 50%, resulting in a false negative rate (FNR) above 50%. The TPR and FNR achieved by the ILR are impractical for retrieving a single binary information bit from the communication established between the T and LR in the ML-RCS. This finding indicates that communication is sufficiently secure for the intended purposes.

### 6.3. Comparative Analysis

The proposed ML-RCS can be compared with the previously proposed RCSs in terms of its performance and data rate. Regarding its performance, we have compared the BER of our system with that of other prominent RCSs mentioned in the literature. Similarly, the data rate has also been analyzed by comparing the number of noise samples (utilized *N*) required to successfully transmit and receive a unit binary digit. Table 5 summarizes the comparison of the proposed ML-RCS with other RCSs mentioned in the literature.

It can be observed that the proposed ML-RCS achieved a BER = 10^−3^ at an MSNR_dB_ = −12, which is the minimum achievable value among all the RCSs. It reflects the proposed ML-RCS’s capability to successfully transmit and receive information under severe channel impairments, which gradually arises when we operate with a lesser MSNR_dB_. Most importantly, the proposed ML-RCS achieved this performance with *N* = 35 only. This reflects that the ML-RCS can transmit more bits (approximately $\frac{2000}{35}$ = 57 more bits) in comparison to the closest competitor, the system of Xu et al. [51], which utilized *N* = 2000 for a unit binary digit. This comparison shows the proposed ML-RCS’s capability to provide better performance with an increased data rate.

## 7. Conclusions

This study presents the first model that incorporates machine learning into RCSs. The proposed ML-RCS uses SkαSNSK to securely transmit binary information from a transmitter within an AWGN channel. The legitimate receiver uses a pretrained DT with an FT configuration to retrieve the binary bits hidden in the received random carrier signals. Our Monte Carlo simulations analyzed the communication perspectives of both the ML and RCSs. Compared with the previously proposed RCSs, the proposed ML-RCS provides a significant increase in the BER performance. The enhanced BER performance of the ML-RCS is governed by the fact that it incorporates ML via the pretrained DT in order to establish secure communication.

In future work, we plan to evaluate different ML algorithms to determine the appropriateness of this task. We are currently investigating the applicability of deploying ML algorithms to improve the RCS performance further. We are in the process of producing RCS architectures incorporated within each of the ML algorithms—SVMs, KNNs, and neural Networks—as separate studies. We have adopted this approach to explore the unique characteristics and contributions of each algorithm when integrated into RCSs. In line with the current approaches to designing RCS architectures, this study initially focused on testing the proposed system in an AWGN channel to establish foundational insights and baseline performance metrics. As it was a standard practice in all previous studies, we tested the proposed ML-RCS in a controlled environment like an AWGN channel only so that a fair comparison could be developed. We are currently inspecting the behavior of the proposed ML-RCS in various channel environments. Subsequent efforts will explore the effectiveness of the ML-RCS in scenarios involving Rayleigh–Rician fading and dynamically varying channels to further evaluate its practical viability. Moreover, incorporating already present or developing secure key distribution methods (independent of pilots) for the proposed system will further ensure the establishment of secure communication. It will prevent interception or tampering during synchronization, leading to a stronger foundation for secure communication systems. With the successful integration of ML into RCSs, we expect that ML algorithms will be utilized in other fields. This will help to increase the level of scientific contributions toward a better and more prosperous society.

## Figures and Tables

**Table 1:** CART classifier.

Configuration	Model Flexibility	Description
FT	Low	Many leaves to make many fine distinctions between classes. (maximum splits = 100)
MT	Medium	Medium number of leaves for finer distinctions between classes. (maximum splits = 20)
CT	High	Few leaves to make coarse distinctions between classes. (maximum splits = 4)

**Table 2:** Parameter settings responsible for LR.

Parameter	Selected Values
α	0.6, 0.8, 1.1
$\beta_{0}$	−0.8, −0.9, −1
$\beta_{1}$	0.8, 0.9, 1
∆β	1.6, 1.8, 2
N	15, 25, 35

**Table 3:** Channel parameters.

Symbol	Definition	Utilized Values
γ	dispersion of noise generated to represent ‘0’ or ‘1’	1
$\gamma_{A}$	dispersion of channel noise	[0.316, 10]
MSNR_dB_	mixed signal-to-noise ratio	[−10, 5]

**Table 4:** Parameter settings responsible for ILR.

Parameter	Selected Values
α	(0, 2]
$\beta_{0}$	[−1, 0)
$\beta_{1}$	[0, 1]
∆β	[0, 2]
N	[1, ∞]

**Table 5:** BER and data rate comparison.

RCSs	Achieved BER = 10^−3^ at MSNR_dB_	Utilized *N*
Cek, M.E. [44]	−6	2000
Ahmed, A.; Savaci, F.A. [45]	−6	1000
Ahmed, A.; Savaci, F.A. [46]	−8	500
Cek, M.E. [47]	−6	1600
Savaci, F.A.; Ahmed, A [48]	−5	1000
Ahmed, A.; Bosnić, Z [50]	−4	1000
Xu et al. [51]	−12	2000
Ahmed, A.; Savaci, F.A. [52]	−5	500
Proposed ML-RCS	−12	35

## Data Availability

The data presented in this study are available on request from the corresponding author.
